# Time Processing in Huntington's Disease: A Group-Control Study

**DOI:** 10.1371/journal.pone.0001263

**Published:** 2007-12-05

**Authors:** Christian Beste, Carsten Saft, Jürgen Andrich, Thomas Müller, Ralf Gold, Michael Falkenstein

**Affiliations:** 1 Department of Neurology, Huntington Centre NRW, St. Josef Hospital, Ruhr-University, Bochum, Germany; 2 Leibniz Research Centre for Working Environment and Human Factors, World Health Organization (WHO) Collaborating Centre for Occupational Health and Human Factors, Dortmund, Germany; 3 Department of Clinical Radiology, University of Münster, Münster, Germany; James Cook University, Australia

## Abstract

**Background:**

“Timing” processes are mediated via a disturbed neuronal network including the basal ganglia. Brain structures important for “timing” are also discussed to be critical for the deterioration of movements in Huntington's disease (HD). Changes in “timing processes” are found in HD, but no study has varied the degree of motor demands in timing functions in parallel in HD. It may be hypothesized that timing functions may be deteriorated to a different extent in motor and non-motor timing, because in motor timing the underlying brain structures may be more demanding than in non-motor timing.

**Methodology/Principle Findings:**

We assessed timing in two different experiments: a time-estimation (TE) and a time-discrimination (TD) task. The demand on motor functions is high in the TE-task and low in the TD-task. Furthermore, general motor ability was assessed at different complexity levels. A presymptomatic (pHD), a symptomatic (HD) and a control group were investigated. We found a decline in timing functions when demands on the motor system were high (TE-task), in HD and even in pHD, compared to controls. In non-motor timing (TD task) and in the assessment of general motor ability, performance in the pHD-group was comparable to the controls and better than in the symptomatic group. Performance in both timing tasks was related to the duration until the estimated age of onset in pHDs.

**Conclusions/Significance:**

The study shows a selective deterioration of time-estimation processes in symptomatic and even presymptomatic Huntington's disease. Time-discrimination processes were not affected in both patient groups. The relation of timing performance to the duration until the estimated age of onset in pHD is of clinical importance.

## Introduction

Huntington's disease (HD) is an autosomal dominant, monogentic neurological disorder causing a degeneration of the neostriatum. The disease is genetically expressed by an extension of the CAG-repeat length at the 4th chromosome [1] encoding a large protein, the “huntingtin”. This protein accumulates and causes apoptotic striatal neuronal death [2]. The most obvious sign of HD is chorea: i.e. rapid, arrhythmic and complex involuntary movements. Especially cortico-striatal circuits [e.g. 3] are affected in Huntington's disease (HD). It is the degeneration of medium spiny neurons (MSPs), which is discussed to be a critical factor in HD [4], [5], but also the dysfunction of the supplementary motor area (SMA) [6] and the dopamine system [7] are important. These brain systems are also affected in the preclinical stage of HD (pHD) [e.g. 3], even though the preclinical stage is characterized by an absence of specific motor signs. Along with the occurrence of irregular dyskinesia, voluntary movements are also deteriorated. Yet the nature of this kind of impairment is not well understood [8], but is of great importance for the patients lives [9].

Optimal motor functioning requires a highly precise timing of the coordination of muscles involved in a movement. If timing functions are crucial, it may be speculated that deficits in cognitive timing functions may underly the voluntary movement disturbances in HD. This seems likely, since “time processing” is mediated via brain systems that are affected in HD and important for movement execution: i.e. cortico-striatal circuits, especially the MSPs, the SMA and the dopamine system [10]–[13]. Indeed, dysfunctions in timing processes are found in HD [e.g. 14], [15], but no study has varied the role of motor demands in timing functions in parallel in HD, to estimate the importance of these processes for voluntary movement dysfunctions in HD. This can be achieved by assessing performance in two different timing tasks: “time-estimation (TE)” and “time-discrimination (TD)”. TE may comprise the “production” of a time interval (e.g. by pressing a button, when a predefined time-period has elapsed). In TD, intervals are defined by stimuli (e.g. tones marking the beginning/ending of the interval) and judged against a standard interval. Thus, precise motor timing is less important in this task, compared to the TE-task (see “Description of Procedures” for more details). Additionally we conducted two other tests assessing general motor performance; a “continuous reaction task (CRT)”, and a “tapping task (TA)” [16] to control effects in motor (TE) and non-motor (TD) embedded timing. If the assessment of timing processes allows an estimation of the integrity of fronto-striatal networks affected in HD, one may assume that performance in these tasks may be related to clinically relevant parameters of disease progression (i.e. the estimated age of onset (eAO) in presymptomatic HD).

In summary, the study aimed at assessing timing processes with varying demands on the motor system in presymptomatic (pHD), symptomatic HD (HD) and healthy controls. Furthermore the relation of possible timing dysfunctions to voluntary movement dysfunctions in HD will be investigated. Finally we want to explore the value of the assessment of timing for the diagnosis of HD.

The results show a selective deterioration of time-estimation processes in presymptomatic Huntington's disease. Time-discrimination processes, as well as general motor performance were not dysfunctional, compared to healthy controls. In the pHD-group performance in the TE and TD-task was related to the estimated age of onset (eAO). In the HD-group, performance was poor in all tasks.

## Methods

### Objectives

The aim of this study was to assess to assess timing processes with varying demands on the motor system in presymptomatic (pHD), symptomatic HD (HD) and healthy controls to estimate if the assessment of timing of timing functions may be of clinical value in the diagnosis of HD.

### Participants

A group of twelve right-handed presymptomatic gene mutation carriers defined by a positive gene test and absence of clinical symptoms (pHD, N = 12) from 22 to 52 years of age (M = 35.91; SD = 10.03) were recruited. The mean CAG-length was 42.58 (SD = 1.78; range = 39–46). Calculation of the estimated age of onset (eAO) [17] revealed a mean eAO of 45.53 (SD = 4.6; range = 37.5–53.2). The duration until estimated age of onset (eAO) was calculated by subtracting the actual age of the patient at time of testing from the calculated eAO and revealed a mean duration of 10.13 (SD = 8.1, range = −3.4–23.8) (Note: negative values in this calculation indicate, that the calculated eAO has already been passed; whereas positive values indicate that the eAO has not passed, according to the estimation by Ranen et al. [17]).

Additionally, a group of ten unmedicated symptomatic gene mutation carries (HD, N = 10) from 21 to 57 years of age (M = 36.50, SD = 10.20) were recruited. The mean CAG-length was 47.10 (SD = 5.4; range = 40–56). The mean age of onset (AO) was 34.20 (SD = 11.5, range = 17–56). All patients (pHD, HD) agreed to be videotaped to document their neurological status. Scores for each group (pHD, HD) of the UHDRS-motor score, UHDRS cognitive score, TFC, IS, MMSE, BDI and YMRS are given in Table 1. Furthermore, a group of twelve age- and gender-matched healthy controls (N = 12) from 23 to 52 years of age (M = 36.5, SD = 8.6) were recruited. See also Table 1 for further details.

**Table 1:** Descriptive demographical and clinical data of the symptomatic group (HD), presymptomatic group (pHD) and control group (controls).

	HD	pHD	Controls
	mean (SD) range	mean (SD) range	mean (SD) range
Sample size	N = 11	N = 14	N = 12
Male∶female ratio	6 m∶5 f	T m∶7 f	6 m∶6 f
Age (years)	36.50 (10.20) 21–57	35.91 (10.03) 22–52	36.50 (8.64) 23–52
CAG-repeat length	47.10 (5.42) 40–56	42.58 (1.78) 39–46	NA
Age of onset (AO)	34.20 (11.51) 17–56	NA	NA
Estimated age of onset (eAO)	**36.99 (11.39) 21–52 (N = 9)**	45.53 (4.62) 37.5–53.3	NA
Duration until eAO	**−0.12 (7.94) −11.5–13.71 (N = 9)**	10.13 (8.19) −3.44–23.82	NA
UHDRS (motor score)	26.00 (12.1) 9–44	0	NA
TFC	11.70 (1.25) 9–13	13	NA
IS	86.50 (8.83) 70–100	100	NA
IQ	105.70 (7.66) 95–118	109.50 (11.86) 95–130	113.25 (8.86) 98–130
UHDRS (cognitive score)	165.40 (24.05) 137–215	236.50 (16.81) 209–259	249.50 (11.03) 236–266
MMSE	27.30 (2.26) 23–30	29.41 (0.51) 29–30	29.66 (0.5) 29–30
BDI	5.90 (4.06) 0–13	5.75 (4.43) 0–14	3.00 (3.43) 0–12
YMRS	3.30 (1.56) 1–5	1.33 (1.37) 0–4	1.36 (1.50) 0–5

### Description of Procedures or Investigations Undertaken

In this study four different tasks were performened. Two tasks were designed to assess timing functions (time estimation (TE); time discrimination (TD), two tasks were designed to assess general motor functions (continuous reaction task (CRT); tapping (TA). The tasks are described in detail, below. The sequence of conducted tests (TE, TD, CRT, TA) was counterbalanced in all groups. All four tasks were trained by the subjects until stable performance was reached.

### Time estimation (TE)

In this task subjects were presented with a white square (2.5×2.5 cm size) on a black computer screen. Subjects were required to press a response key as exactly as possible at 1200 ms after onset of a stimulus (a square). This time, which includes the estimated time for the motor response, had to be estimated by the subjects, i. e. the motor response defines the length of the time interval. Feedback was given if the response was made in a time range from 1000–1400 ms or not. If the response was made within that time range, positive feedback was given. If not, negative feedback was given; 120 trials were conducted.

### Time discrimination (TD)

Time discrimination was assessed using a two-alternative-forced-choice paradigm. At the beginning of this task white letters “S” and “L” (2.5×2.5 cm size) were presented on a black computer screen. The stimuli differed in their duration of presentation. “S” was presented for 1000 ms and “L” was presented for 1200 ms. The subjects had to react with the left hand on “S”-presentations and with the right hand on “L”-presentations. After the subjects were familiar with the different presentation times of “S” and “L” they underwent 120 trials, in which the letter “H” was presented instead of “S” and “L”. The presentation times of “H” randomly changed between 1000 ms and 1200 ms (60 trials 1000 ms; 60 trials 1200 ms) and the subjects had to decide using the appropriate response, if the presentation of the letter “H” was presented for a short (i.e. 1000 ms) or long period (i.e. 1200 ms). This decision had to be carried out *after* the stimulus was presented. Thus the time interval is not defined by a precise motor response, making timing not as heavily dependent on precise motor function as in the TE-task.

### Continuous reaction task (CRT)

In this task subjects were presented with white arrowheads (2 cm horizontal and vertical size), either pointing to the left or to the right. The subjects were required to react with the left hand when the arrowhead pointed to the left and with the right hand when the arrowhead pointed to the right. 60 arrowheads pointed in the left and 60 pointed to the right direction. The subjects were required to respond as fast as possible.

### Tapping (TA)

This task assesses simple movements [16]. Subjects were instructed to tap as quickly as possible on a computer-based contact board (3×3 cm) with a contact pencil for a period of 32 seconds after the initial flash of a yellow stimulus light. The board was positioned in the centre. When performing the task, elbows were allowed to be in contact with the table. The number of contacts was measured [16]. For statistical analysis performance the absolute frequency of taps was collapsed over the left and right hand. Additionally, the intertap-interval was calculated by dividing the time period of tapping (64 seconds for both hands) by the absolute frequency of performed taps.

### Ethics

Asymptomatic gene carriers as well as symptomatic patients were recruited from the local Huntington Centre of the Neurological Clinic of the University of Bochum. They were selected on the basis of pre-clinical counselling in the case of asymptomatic gene mutation carries or on the basis of routine clinical counselling in the case of symptomatic patients. Healthy controls were recruited by newspaper announcements. All participants gave written informed consent. For the symptomatic individuals, a family member was aware of the recruitement for the study and was involved in the consent procedure. The study was approved by the ethics committee of the University of Bochum.

### Statistical methods

Data from twelve presymptomatic gene mutation carriers (pHD) (N = 12), ten symptomatic subjects (HD) (N = 10) and twelve healthy controls (N = 12) was analyzed. There were no drop-outs. For the TE- and TD-task the number of error trials was analyzed. For the CRT-task the mean reaction time across all trials was analyzed. For the tapping task (TA) the mean number of performed taps as well as the intertap-interval was analyzed. Tests of normal distribution within each group using the Kolmogorov-Smirnov test revealed that each variable included to the ANOVAs and correlational analysis was normal distributed (all z<0.877; *P* >.425; one-tailed). The TE- and TD-task were analyzed in a repeated-measures ANOVA using the within-subject factor “experiment” (TE vs. TD) and the between-subject factor group (controls, pHD, HD). Both the CRT- as well as the tapping task (TA) were analyzed using separate univariate ANOVAs with the between-subject factor group (controls, pHD, HD). The degrees of freedom were adjusted using the Greenhouse-Geisser-Correction when appropriate. Significances are given one-tailed, due to higher test-power. In addition effect sizes (η) are reported. The mean and standard deviation (±SD) are given. Post-hoc tests were calculated using the Bonferroni-correction. Due to higher test power, one-sided tests were performed. For statistical analysis SPSS 11.0 was used.

## Results

**Figure 1:**
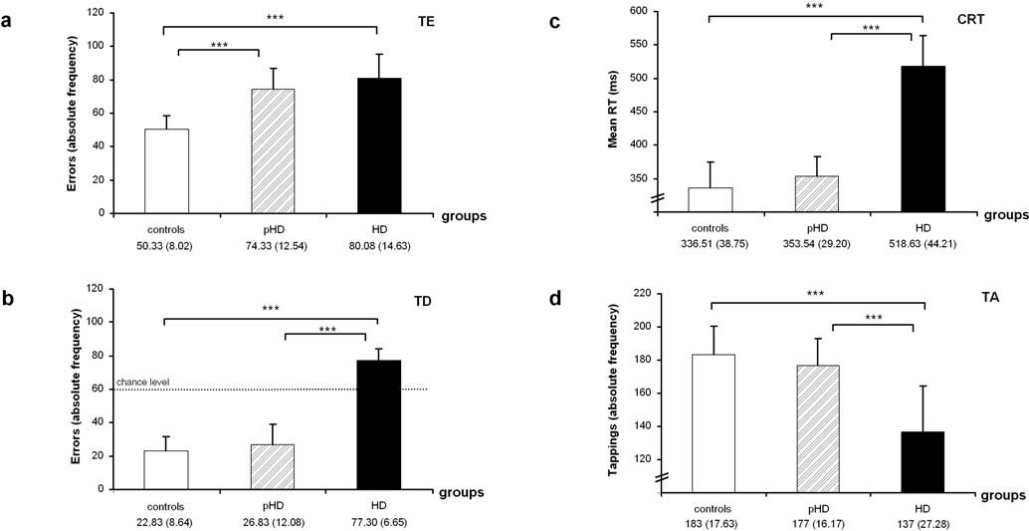
Performance on the TE (a), TD (b), CRT-task (c) and tapping (d). In (a) and (b) the absolute frequency of errors is given (note: In TD, performance differed from chance level), in (c) the mean reaction time, in (d) the number of taps are given, all with standard deviation (SD).

Results of the performance in each test are given in Figure 1. Analyzing the TE- and TD-task, the main-effect “group” (F(2,31) = 66.40; *p*<.001; η = .81) showed that the number of errors increased from controls (36.58±16.23) to pHD (50.58±27.08) and HD (79.05±11.20). The main-effect “experiment” (F(1,31) = 166.29; *p*<.001; η = .84), showed poorer performance (i.e. more errors) in the TE-task (68.48±17.60) than in the TD-task (40.26±26.02). Both effects interacted with each other (F(1,30) = 37.05; *p*<.001; η = .70). Subsequent univariate ANOVAS revealed a group-effect in both tasks (TE: F(2,31) = 20.83; *p*<.001; η = .57; TD: (F(2,31) = 128.01; *p*<.001; η = .89). Post-hoc tests for the TE-task showed that controls (50.33±8.02) performed better than both HD-groups (pHD: 74.33±12.54; HD: 80.08±14.63) (*p*<.001), which did not differ from each other (*p* = .637) (Fig. 1a). For the TD-task pHDs and controls did not differ from each other (pHD: 26.83±12.08; controls: 22.83±8.64) (*p* = .461), and both performed better than the HD-group (77.30±6.65) (*P*<.001) (Fig. 1b). For the TD-task, performance was tested against chance level and it was shown that in each group performance was different from chance level (controls: t_12_ = −14.89 *p*<.001; pHD: t_12_ = −10.68 *p*<.001; HD: t_10_ = 8.22 *p*<.001; all one-tailed).

The CRT-task also revealed a group-effect (F(2,31) = 21.59; *p*<.001; η = .58) with the pHDs and controls not differing from each other (controls: 336.5±38.7; pHD: 353.5±29.2) (*p*>.9), but performing better than the HD-group (518.6±44.21) (*p*<.001) (Fig. 1c). The same pattern was also found for the absolute frequency of taps in the TA-task (Fig. 1d) (F(2,31) = 15.55; *p*<.001; η = .50) with pHDs and controls not differing from each other (controls: 183±17.6; pHD: 177±16.1) (*p* >.9), and performing better than the HD-group (137±27.2) (*p*<.001). For the calculated inter-tap interval there was the same effect than for absolute frequencies (F(2,31) = 19.45; p<.001) with the pHDs and controls not differing from each other (controls: 352.46±15.11; pHD: 363.39±15.13) (p>.9) and performing better than the HD-group (480.43±16.55) (p<.001).

**Figure 2:**
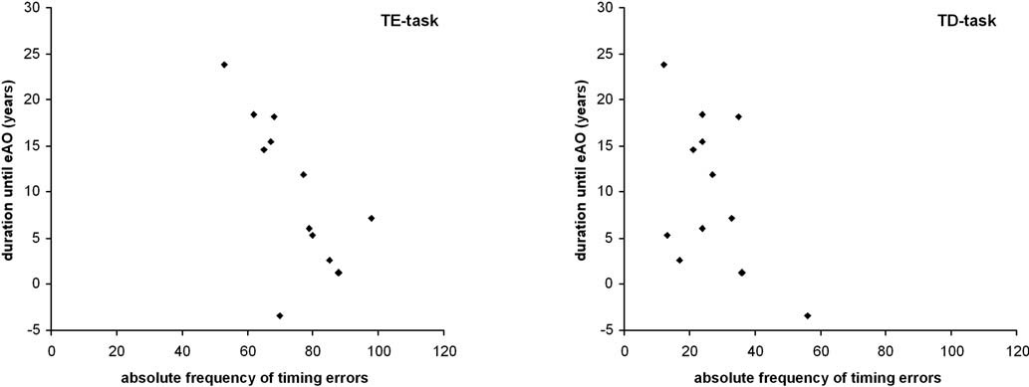
Relation of the performance in the TD and TE task to the duration until the estimated age of onset.

In pHDs performance in the TE and the TD tasks was related to the duration until the eAO in the pHDs [17] (TE: r = −.689, *P* = .007; TD: r = −.502, *p* = .048) (refer: Figure 2), while performance in CRT (r = −.089, *p* = .391), tapping (absolute taps: r = −.200, *p* = .266; intertap-interval: r = −.172, p = .279) was not.

In sum, both HD-groups showed comparable performance in motor embedded timing (TE) and performed more poorly than the control group, while in TD controls and pHDs showed comparable performance [see: 15], which were better than those of HD-group. The latter pattern was also found in the CRT- and TA-task.

## Discussion

The results suggest that in presymptomatic gene mutations carriers (pHD) performance in the TE-task is deteriorated, while performance in the TD-, CRT- and TA-task is still normal. Additionally, performance in the timing tasks (i.e. TE- and TD-task) was related to the estimated age of onset (eAO) in pHD. For the symptomatic group (HD) performance was generally poor.

The result found for the TD-task is in line with the findings by Paulsen et al. [15], who also reported no difference in behavioural performance in this task, likely due to compensatory mechanisms [15]. Yet data from the TE-task reveal that performance can decline, even in pHD, if timing is embedded in motor processes. The difference in results regarding the TD- and the TE-task suggests that timing is not per se deteriorated in pHD, but it becomes when timing is dependent on motor processes. Yet, if HD wasa “time-perception” disorder, the pHD-group should have performed poorly on both timing tasks, which was not the case. If HD was a “motor timing” disorder performance in the CRT- and TA-task may be different in the pHD-group, since motor timing is also important in these tasks. This was likewise not the case. However, performance in the TE-task was poor in pHDs, suggesting for “motor timing” deficits. The critical point that may dissociate the CRT- and TA-task from the TE-task is that in the latter task, timing was at a larger timescale than in the CRT- and TA-task. In the TE-task intervals of 1200 ms had to be produced. Mean reactions times (RTs) in the CRT were more than twice as short. This was also the case for the mean intertap-interval in the TA-task. Therefore we assume that the motor timing deficit in pHD depends on the length of the time interval. Future studies should examine different time intervals in motor timing tasks in order to determine the optimal length for detecting early changes in HD.

### Possible mechanisms

Regarding the “neuroanatomy of timing” striatal medium spiny neurons (MSPs), the dopamine system and the SMA play a central role in a network mediating time control [11], [12]. Here medium spiny neurons (MSPs) are most central. MSPs are assumed to weight cortical and thalamic inputs, which carry “time-information” from cortical neurons [11]. In cortical structures time information is represented by neurons oscillating in activity at a different rate [11]. These various activities impinge on striatal MSPs. The assumed weighting functions of the MSPs are assumed to serve as a filter mechanism through which coherent cortical activity can elicit striatal firing only at the appropriate time. However, besides the putative function of the MSPs for timing functions, they are also important for execution of movements. As stated in the introduction MSPs are affected in HD [5], [6], [7] and assumed to relate to the appearance of motor dysfunctions [5], [6]. It is therefore hypothesized that in case of time estimation (TE) the weighting processes mediated via the MSPs may interfere with the concurrently occurring preparation of the voluntary movement also partly mediated via the MSPs. Due to this putative coincidence of weighting processes and motor preparation processes the capacity of the MSPs may become overstrained in pHDs leading to a decline in performance. In HD it is likely that the capacity of the common part of the systems is reduced to an extent that even “less demanding” processes [i.e. non-motor timing (TD), motor performance (CRT, TA)] are performed poorly. This is plausible since progressive neuropathology is evident even in pHD [3], [18]. However, the preceding discussion focussed on the MSPs, but it should be noted that also the dysfunctional dopamine system [3] and the dysfunctional SMA [6] in HD play an important role in timing.

### Correlational data

Despite performance in all tasks being poor in HD, only performance in timing-tasks was inversely related to the duration until the estimated age of onset (eAO) in pHDs [see: 15]. That is: the nearer the eAO, the poorer was performance in the TE- as well as in the TD-task. Thus, the estimated forthcoming time span of preclinical disease progression, culminating in the onset of movement dysfunctions (age of onset, AO) is related to alterations in timing-processes, with these processes being more and more affected the closer the eAO. This is underlined by the fact that a relation to the duration until eAO was seen in both timing tasks and thus independent of the demands on motor functions. Due to this it may be speculated that “timing-processes” and their deterioration may be another putative origin for the emergence of motor symptoms in HD, besides alterations in error processing [19], which have indeed been shown to be deficient in HD [20]–[22].

### Limitations

Future studies may incorporate larger sample sizes in order to increase generalizability of results. What may also be critical in the current study is, that CAG-repeat sizes differed between the presymptomatic (pHD) and symptomatic group (HD), since different CAG-repeat size may be accompanied by different strengths of pathogenic processes. Yet, this was side effect of matching the groups with respect to their age, since tasks assessing cognitive timing functions are known to be age-sensitive. Since this study was restricted to behavioural data, the precise brain mechanisms underlying the observed pattern of results must remain hypothetical.

### Perspectives and Conclusion

In summary, the study shows a selective deterioration of time-estimation processes in presymptomatic Huntington's disease. Time-discrimination processes were not affected. It may be inferred that under special circumstances voluntary movements can be deteriorated even in the preclinical stage of HD. These may depend on the time-scale in which the movement takes place. Furthermore, performance in the TE- and TD-task is related to the duration until the likely onset of motor symptoms in HD. Future studies may be done in a longitudinal manner to better document the time course of preclinical disease progression and strengthen clinical importance. This may also be an important step in order to estimate if these easy and fast applicable tasks may be implemented in large clinical trials.

## References

[1] No authors listed (1993). A novel gene containing a trinucleotide repeat is expanded and unstable on Huntington's disease chromosomes. The Huntington's Disease Collaborative Research Group.. Cell.

[2] Paulsen JS, Zhao H, Sout JC, Brinkman RR, Guttman M (2001). Clinical markers of early disease in persons near onset of Huntington's disease.. Neurology.

[3] Rosas HD, Feigin AS, Hersch SM (2004). Using advances in neuroimaging to detect, understand, and monitor disease progression in Huntington's Disease.. NeuroRx.

[4] Cepeda C, Wu N, Andre VM, Cummings DM, Levine MS (2007). The corticostriatal pathway in Huntington's Disease.. Prog Neurobiol.

[5] Mitchell IJ, Cooper AJ, Griffiths MR (1999). The selective vulnerability of striatopallidal neurons.. Prog Neurobiol.

[6] Bartenstein P, Weindl A, Spiegel S, Boecker H, Wenzel R (1997). Central motor processing in Huntington's Disease. A PET study.. Brain.

[7] Kendall AL, Hantraye P, Palfi S (2000). Striatal tissue transplantation in non-human primates.. Prog Brain Res.

[8] Kremer, Bates G, Harper P, Jones L (2002). Clinical Neurology of Huntington's Disease.. Huntington's Disease.

[9] van Vugt JP, Piet KK, Vink LJ, Siesling S, Zwinderman AH (2004). Objective assessment of motor slowness in Huntington's disease: clinical correlates and 2-year follow-up.. Movement Disorders.

[10] Buhusi CV, Meck WH (2005). What makes us tick? Funtional and neural mechanisms of interval timing.. Nat Rev Neurosci.

[11] Matell MS, Meck WH (2004). Cortico-striatal circuits and interval timing: coincidence detection of oscillatory processes.. Brain Res Cogn Brain Res.

[12] Macar F, Coull J, Vidal F (2006). The supplementary motor area in motor and perceptual time processing: fMRI studies.. Cogn Process.

[13] Coull JT, Vidal F, Nazarian B, Macar F (2004). Functional anatomy of the attentional modulation of time estimation.. Science.

[14] Hinton SC, Paulsen JS, Hoffmann RG, Reynolds NC, Zimbelman JL (2007). Motor timing variability increases in preclinical Huntington's Disease patients as estimated onset of motor symptoms approaches.. J Int Neuropsychol Soc.

[15] Paulsen JS, Zimbelman JL, Hinton SC, Langbehn DR, Leveroni CL (2004). fMRI biomarker of early neuronal dysfunction in presymptomatic Huntington's Disease.. AJNR Am J Neuroradiol.

[16] Saft C, Andrich J, Meisel NM, Przuntek H, Müller T (2006). Assessment of simple movements reflects impairment in Huntington's Disease.. Movement Disorders.

[17] Ranen NG, Stine OC, Abbott MH, Sherr M, Codori AM (1995). Anticipation and instability of IT-15 (CAG)n repeats in parent-offspring pairs with Huntington's disease.. Am J Hum Genet.

[18] Aylward EH, Sparks BF, Field KM, Yallapragada V, Shpritz BD (2004). Onset and rate of striatal atrophy in preclinical Huntington disease.. Neurology.

[19] Smith MA, Brandt J, Shadmehr R (2000). Motor disorder in Huntington's disease begins as a dysfunction in error feedback control.. Nature.

[20] Beste C, Saft C, Yordanova J, Andrich J, Gold R (2007). Compensation or pathology in cortico-subcortical interactions in preclinical Huntington's Disease?. Neuropsychologia.

[21] Beste C, Saft C, Konrad C, Andrich J, Habbel A (2007). Levels of error processing in Huntington's disease: a combined study using event-related potentials and voxel-based morphometry.. Hum Brain Mapp [Epub ahead of print].

[22] Beste C, Saft C, Andrich J, Gold R, Falkenstein M (2006). Error processing in Huntington's Disease.. PLoS One.

